# MicroRNA: a prognostic biomarker and a possible druggable target for circumventing multidrug resistance in cancer chemotherapy

**DOI:** 10.1186/1423-0127-20-99

**Published:** 2013-12-20

**Authors:** Kenneth KW To

**Affiliations:** 1 School of Pharmacy, Room 801 N, Lo Kwee-Seong Integrated Biomedical Sciences Building, Faculty of Medicine, The Chinese University of Hong Kong, Area 39, Shatin, New Territories, Hong Kong SAR, China

**Keywords:** ATP-binding cassette transporters, microRNA, Multidrug resistance, Non-coding RNAs, Prognostic biomarkers, 3′ untranslated region

## Abstract

Multidrug resistance (MDR) is a major obstacle to successful cancer treatment. It is often associated with an increased efflux of a variety of structurally unrelated anticancer drugs by ATP-binding cassette (ABC) transporters including P-gp, ABCG2 and MRP1. MicroRNAs (miRNAs) are small non-coding RNAs that govern posttranscriptional regulation of target genes by interacting with specific sequences in their 3′ untranslated region (3′UTR), thereby promoting mRNA degradation or suppressing translation. Accumulating evidence suggests that alterations in miRNAs contribute to resistance to anticancer drugs. While miRNAs are well-known to be dysregulated in cancer, recent literature revealed that miRNA levels in biological samples may be correlated with chemotherapy response. This review summarized the coordinated network by which miRNA regulated MDR transporters. The usefulness of miRNAs as prognostic biomarkers for predicting chemotherapeutic outcome is discussed. MiRNAs may also represent druggable targets for circumvention of MDR.

## Introduction

Resistance to anticancer drugs remains a major unresolved obstacle to successful chemotherapy. It has been estimated that most cancer deaths, if not all, are caused by chemotherapy failure because tumors quickly develop resistance after exposure to drugs [1]. In order to develop novel strategies to combat cancer drug resistance and to improve patient survival, a thorough understanding of its mechanisms is therefore badly needed. The causes of cancer drug resistance are multifactorial, including decreased accumulation/increased disposition of anticancer drugs, mutation of drug targets, enhanced cell repair and altered cell death pathways. However, the most common and extensively studied mechanism is the overexpression of the energy-dependent ATP-binding cassette (ABC) drug efflux transporters such as P-glycoprotein (MDR-1/P-gp/ABCB1), multidrug resistance related protein (MRP-1/ABCC1), and breast cancer resistance protein (BCRP/MXR/ABCP/ABCG2) [2]. It is associated with an increased efflux of cytotoxic drugs, causing multidrug resistance (MDR) because cytotoxic drugs from different chemical structures are affected simultaneously.

MicroRNAs (miRNAs) are short endogenous non-coding RNAs that repress gene expression in a variety of eukaryotic organisms. Gene regulation by miRNAs is mediated by the formation of imperfect hybrids with the 3′untranslated region (3′UTR) sequences of the target mRNAs, leading to mRNA degradation and/or translational inhibition [3]. They play important roles in several cellular processes, such as proliferation, differentiation, apoptosis, and development, by simultaneously controlling the expression level of hundreds of genes. MiRNAs are predicted to regulate the expression of up to one third of human protein-coding genes [4-6].

Numerous recent studies have shown that miRNA expression profiles differ between normal tissues and cancerous cells derived from the same organ, and also between cancer types [7]. MiRNAs can act as oncogenes or tumor suppressors, contributing to different pathways in tumorigenesis [8,9]. They may be used for diagnostic and prognostic purposes and they also constitute novel targets for cancer treatment [10,11]. Recently, the evidence for the roles of miRNAs in determining drug sensitivity/resistance has been emerging. This review summarized the current understanding about the role of miRNAs in mediating cancer drug resistance. More emphasis is placed on miRNA-related regulation of the MDR transporters, though other mechanisms causing drug resistance not related to transporters will also be discussed. The possible application of miRNA-transporters regulatory network for predicting chemotherapeutic response will be highlighted. Novel strategies aiming to target miRNA-related pathways for the circumvention of multidrug resistance will also be elaborated.

## Review

### Aberrant expression of miRNAs and cancer drug resistance

Evidence pointing to the role of miRNAs in determining drug sensitivity and MDR is emerging. MiRNA expression is largely dysregulated in drug-resistant cancer cells [12,13]. In a recent study on a doxorubicin-resistant breast cancer cell line MCF-7/DOX, a profound dysregulation of the miRNA profile and altered expression of two important miRNA processing enzymes Dicer and Argonaute 2 was reported [14]. The remarkable correlation between specific miRNA expression and the corresponding changes in protein levels of their specific targets having well-documented role in cancer drug resistance, may thus implies a mechanistic link between miRNAome dysregulation and the MDR phenotype. Moreover, miRNA expression patterns in the NCI-60 drug screen cell lines are significantly correlated to the sensitivity patterns of the cancer cells for a variety of anticancer drugs [15]. Furthermore, numerous miRNAs have been found to regulate drug resistance genes such as *ABCG2*[16-18], *BCL2*[19], *DHFR*[20], *MDR1*[14] and *PTEN*[21]. Importantly, modulation of miRNA expression or function can alter sensitivity of cancer cells to anticancer drugs. This could be achieved by inhibiting the function of up-regulated miRNAs or restoring the expression of down-regulated miRNAs. Together, miRNAs may represent key players in both intrinsic and acquired MDR in cancer cells [22].

The cause of cancer drug resistance is multifactorial. The role of miRNAs in mediating cancer drug resistance is separately discussed below according to whether they are regulating to (I) MDR transporters-mediated (Table 1); or (II) non-MDR transporters-mediated mechanisms (Table 2). The list is by no means exhaustive. We aim to illustrate the representative ones with potentially wider implications.

**Table 1:** miRNAs reported to regulate MDR transporters to mediate chemoresistance

**Transporter targets**	**MicroRNA dysregulation**	**Direct binding site(s) on target gene?**	**Prediction by miRNA database(s)**	**Identification method**	**Cancer type**	**Study in patient samples?**	**Reference**
ABCB1	miR-27a↑	*Indirect regulation*-targets HIPK2 (a transcriptional corepressor that inhibits HIF-1αactivity), thereby indirectly activating MDR-1/P-gp	TargetScan suggests that HIPK2 is a possible target for miR-27a	Serendipitous testing of miR-27a in the development of drug resistance in ovarian cancer cell lines	Ovarian	No	[23]
	miR-27a↑, miR-451↑	*Likely indirect regulation*	--	miRNA microarray profiling	Ovarian	No	[24]
		*(i.e. not via ABCB1 3′UTR)*					
		miR-27a & miR-451 mimics elevate ABCB1 mRNA					
	miR-122↓	NO attempt to verify binding of miR-122 on MDR-1 3′UTR	NOT performed	Serendipitous testing of downregulation of MDR related genes (MDR-1, MRP & GST-π) by miR-122	Hepatocellular carcinoma	No	[25]
	miR-296↑	*Likely indirect regulation*	--	miRNA microarray profiling	Esophageal squamous cell carcinoma	Yes	[26]
		*(i.e. not via ABCB1 3′UTR)*					
		- antagomir of miR-296 decrease ABCB1 promoter activity					
	miR-298↓	Confirmed by luciferase reporter assay / MDR-1 3′UTR miRNA site deletion / miRNA mimic / miRNA inhibitor	miRanda	miRNA microarray	Breast cancer	No	[27]
	miR-1253↓						
	let-7 g↓	*Indirect regulation*			Ovarian	Yes	[28]
		• targets IMP-1 (a RNA-binding protein)					
		• IMP-1 is known to stabilize MDR1 mRNA					
		• loss of let-7 g commonly observed in various cancers could therefore allow overexpression of IMP-1 and stability of MDR-1/P-gp to mediate drug resistance					
ABCC1	miR-326↓	Confirmed by luciferase reporter assay / ABCC1 3′UTR miRNA site deletion / gene expression analysis after transfection with miR-326 mimic	TargetScan	miRNA microarray profiling	Breast cell line + early/advanced breast cancer tissue	Yes	[29]
	miR-1291↓	Confirmed by luciferase reporter/miRNA mimic/miRNA inhibitor	RNAhybrid, TargetScan, miRanda, PITA	Serendipitous study of the role of SNORA34 to generate miR-1291, which subsequently controls chemosensitivity	Pancreatic	No	[30]
ABCC2	miR-297↓	Luciferase reporter/miRNA mimic/miRNA inhibitor	TargetScan	miRNA microarray profiling	Colorectal	Yes	[31]
ABCC3 & ABCC6	miR-9*↓	*Indirect regulation* –	PicTar, TargetScan, miRBase, miRanda	Serendipitous study of the role of ID4 in chemoresistance of induced glioma stem cells	Glioma	Yes	[32]
		• miR-9* is a SOX2-targeting miRNA					
		• novel ID4-miR-9*-SOX2-ABCC3/ABCC6 regulatory pathway					
		• ID4 was found to confer chemoresistance to glioma stem cells by inducing the expression of two SOX2-mediated ABC transporters (ABCC3 & ABCC6) through suppression of miR-9*					
ABCG2	miR-212↓, miR-328↓	Effect of miR-212 & miR-328 on ABCG2 expression was evaluated by gene expression analysis and luciferase reporter gene assay	--	miRNA microarray profiling in imatinib-selected K562 cells	Leukemia (short-term /long-term imatinib treatmet)	No	[33]
	miR-328↓	Confirmed by luciferase reporter assay / ABCG2 3′UTR miRNA site deletion / miRNA mimic / miRNA antagomir	PITA; TargetScan	Bioinformatic analysis	Breast	No	[18]
	miR-328↓	Confirmed by luciferase reporter assay / gene expression analysis after transfection with miRNA mimic or inhibitor	--	miRNA microarray analysis on SP^#^ vs non-SP^#^ cells	Colorectal cell lines + primary biopsies	Yes	[34]
	miR-328↓, miR-519c↓, miR-520 h↓	Confirmed by luciferase reporter assay with site-directed gene mutagenesis / gene expression analysis after transfection with miRNA mimics	TargetScan, PITA, MicroCosm Targets, RNA22	Bioinformatic analysis	Breast, Stem-like cells from human retinoblastoma	No	[35]
	miR-519c (shortening of ABCG2 3′UTR escape miR-519c repression)	Confirmed by luciferase reporter assay with site-directed gene mutagensis on ABCG2 3′UTR miRNA site / gene expression analysis after transfection with miRNA mimics or inhibitor	miRBase TARGETS, RNAHybrid, UTRScan program	Bioinformatic analysis	Colon	No	[16,17]
	miR-520 h↓	Confirmed by luciferase reporter assay +/- miRNA mimic & inhibitor	PicTar, miRanda, TargetScan	miRNA microarray profiling of CD34+ hematopoietic cells	Leukemia	No	[36]
	miR-520 h↓	Confirmed by gene expression analysis after miRNA mimic transfection	miRanda, TargetScan, TarBase	Bioinformatic analysis	Pancreatic	No	[37]
	miR-181a↓	Confirmed by luciferase reporter assay and gene expression analysis +/- miRNA mimic & inhibitor	RNAhybrid	miRNA microarray analysis to compare sensitive and resistant cells	Breast	No	[38]
	miR-487a↓	Confirmed by luciferase reporter assay and gene expression analysis +/- miRNA mimic & inhibitor Confirmed by luciferase reporter assay and gene expression analysis +/- miRNA mimic & inhibitor	TargetScan, PITA, RNAhybrid	Bioinformatic analysis	Breast	No	[39]

^#^ SP = side population; Non-SP = non-side population.

**Table 2:** miRNAs reported to regulate other mediators of drug resistance

**Target gene**	**Biological effect of target gene**	**MicroRNA**	**Validation of miRNA binding site**	**Prediction by miRNA database(s)**	**Identification method**	**Cancer type/Significance/Specific type of drug resistance**	**Study in patient samples?**	**Reference**
*BAX*	Pro-apoptotic	miR-296↑	Luciferase reporter assay + miRNA antagomir	--	miRNA microarray profiling	Esophageal squamous cell carcinoma	Yes	[26]
*BCL2*	Anti-apoptotic	miR-15b↓	Luciferase reporter assay + site-driected mutagenesis of Bcl-2 3′UTR + miRNA mimic	miRBase & TargetScan	miRNA microarray profiling	Gastric	No	[19]
		miR-16↓						
*BCL2*	Anti-apoptotic	miR-1915↓	Luciferase reporter assay + site-directed mutagenesis of Bcl-2 3′UTR + miRNA mimics & inhibitors	miRDB; TargetScan (4 possible miRNA binding sites within Bcl-2 3′UTR)	miRNA microarray profiling	Colorectal	No	[40]
*BCL2 & SIRT1*	Anti-apoptotic	miR-34a↓	*Direct miRNA effect* (on Bcl-2 & SIRT1 mRNA): Luciferase reporter +/- miRNA mimic	TargetScan	Mechanistic investigation of miR-34a/Bcl-2 and miR-34a/SIRT1 pathways in taxane-based chemotherapy	Prostate / Paclitaxel resistance	No	[41]
			*Indirect miRNA effect* (through HuR): Gene expression analysis +/- miRNA mimic					
*BMI-1*	PcG^a^ protein – transcriptional repressor	miR-200c↓	Gene expression analysis after infection with miR-200c vector	--	miRNA microarray analysis	Melanoma /miR-200c is commonly found to be downregulated in malignant melanoma that possess self-renewal cancer stem-cell like property and are more invasive. The prominent miR-200c downregulation is accompanied by Bmi-1 overexpression, which was found to cause loss of E-cadherin (thereby EMT) and overexpression of MDR transporters (including ABCG2, ABCG5 and MDR).	Yes	[42]
*CDH1*	EMT^b^ transition	mR-200c↓	*Indirect regulation:* The two miRNAs repress ZEB1 & ZEB2, thereby leading to loss of E-cadherin and a EMT phenotype	--	Mechanistic investigation of the role of miRNAs in EMT-linked docetaxel resistance in prostate cancer	Prostate/Docetaxel resistance/Docetaxel treatment triggers EMT to inhibit apoptosis through the proposed miR-200c/205/ZEB1/ZEB2/E-cadherin pathway	No	[43]
		miR-205↓						
*DHFR*	Folate metabolism	miR-24 (DHFR SNP 829C > T makes DHFR mRNA indifferent to miR-24)	Gene expression analysis + Site-directed mutagenesis + miRNA mimics & inhibitors	MiRanda & miRBase	A 829C > T SNP identified in *DHFR* gene in Japanese population was found to be associated with increased DHFR message	Fibrosarcoma/methotrexate resistance	No	[20]
*HIPK2*	Tumor suppressor	miR-27a↑	NO attempt to verify binding of miR-27a on HIPK2 3′UTR	TargetScan	Identified during the study of indirect effect of miR-27a on MDR-1 expression	Ovarian	No	[23]
*PTEN*	Tumor suppressor	miR-214↑	Luciferase reporter assay + site-driected mutagenesis of PTEN 3′UTR + miRNA mimic	--	miRNA microarray profiling	Ovarian/cisplatin resistance	Yes	[21]
*TUBB3*	Structural protein (β-tubulin)	miR-200c↓	Gene expression analysis +/- miRNA mimic	--	miRNA microarray profiling	Resistance to microtubule –binding chemotherapeutic drugs	No	[44]

^a^ PcG = polycomb group protein (transcriptional repressor).

^b^ EMT = epithelial-to-mesenchymal transition.

## Regulation of ABC transporters-mediated MDR by miRNAs

### Direct regulation by miRNAs

#### ABCG2 (also known as BCRP/MXR/ABCP)

ABCG2 is the first MDR transporter reported to be regulated by miRNA-mediated mechanism. It is one of the major ABC transporters contributing to the MDR phenotype. Overexpression of the *ABCG2* gene is frequently observed in cancer cell lines selected with chemotherapeutic drugs [2,45]. To date, most studies examining the regulation of ABCG2 have focused on transcription. Gene amplification, chromosome translocation, and the use of alternative 5′ promoters due to differential expression of splice variants at the 5′-untranslated region (5′UTR) of ABCG2 mRNA have been reported to play important roles in the increased expression of ABCG2 [46,47]. In contrast, the understanding about posttranscriptional regulation of ABCG2 has just started to evolve.

To date, a few miRNAs (miR-520 h [36,37], -519c [16,17], -328 [18], -181a [38], & -487a [39]) have been identified by different research groups independently to regulate ABCG2 expression by interacting directly with ABCG2 3′UTR and to determine the sensitivity of cancer cells to chemotherapeutic drugs (Table 1). Consistent with the hypothesis that aberrant miRNA expression can cause cancer drug resistance, low miR-328 expression was found to correlate with the overexpression of ABCG2 in resistant MCF-7/MX100 breast cancer cells [18]. In a human retinoblastoma cell line model, it has been further demonstrated that low expressions of all three miRNAs (miR-328, -519c, & -520 h) correlate very well with high ABCG2 expression, with concomitant expression of other stem cell markers including CD133 and ALDH1A1 [35]. On the other hand, miR-520 h has been reported to promote differentiation of hematopoietic stem cells by inhibiting ABCG2 expression [36]. ABCG2 has been suggested to be a survival factor for stem or cancer stem cells (CSCs) [48]. Thus these findings collectively support an important role played by miRNAs in maintaining high ABCG2 level in CSCs, leading to drug resistance. It will be interesting to verify if the same phenomenon is also observed in tumor samples from patients not responding to cancer chemotherapy.

##### Drug-resistant cancer cells escape miR-519c-mediated ABCG2 repression by shortening of ABCG2 mRNA 3′UTR

**Figure 1:**
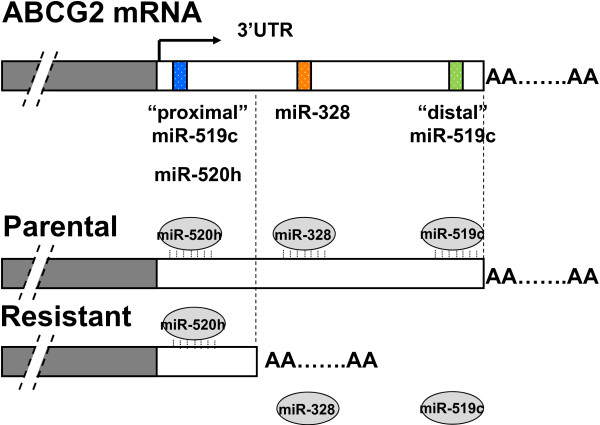
**A proposed model for ABCG2 regulation by miR-519c.** ABCG2 mRNA adopts predominantly a long form of 3′UTR in parental S1 cells but only the shorter forms in drug-resistant S1M1-80 cancer cell line [16,17]. MiR-519c (and miR-328) can only bind to the long ABCG2 3′UTR in parental cells, thus allowing ABCG2 in resistant cells to escape from their repression and thereby acquiring a higher expression.

An additional layer of complexity in miR-519c-mediated regulation of ABCG2 has been proposed, which is associated with alternative cleavage and polyadenylation of the 3′UTR of ABCG2 mRNA, to facilitate the drug resistance phenotype [17]. ABCG2 mRNA was found to be more stable in drug-selected and ABCG2-overexpressing resistant cell lines than in their parental counterparts [16,17]. Given that the expression level of miR-519c in the parental and resistant cells does not differ too much in the cell line model studied, the increase in mRNA stability was subsequently tied to a missing miR-519c binding site in the truncated 3′UTR of ABCG2 mRNA in drug resistant cells [17]. MiR-519c cannot bind to ABCG2 mRNA in the resistant cells because of the shorter 3′UTR, and thus miRNA-mediated mRNA degradation and/or protein translation block are relieved, contributing to ABCG2 overexpression (Figure 1).

In eukaryotes, polyadenylation is an important process that produces mature mRNA for translation. A poly(A) tail is added to 3′ end of mRNA at the end of transcription, which protects the mRNA molecule from enzymatic degradation in the cytoplasm, aids in transcription termination, export of the mRNA from nucleus, and translation [49]. Importantly, alternative polyadenylation can occur when several polyA signals lie in the last exon of a protein-coding gene, generating mRNA isoforms with different 3′UTR length. While the phenomenon of alternative cleavage and polyadenylation has been known for decades, it has only been fully appreciated recently [50]. The majority of human genes contain at least two polyA sites [51], and alternative cleavage and polyadenylation has been shown to be fairly common in multiple organisms [50]. In general, proliferative cells, such as induced pluripotent stem cells and cancer cells, show a global shortening of 3′UTR as compared with their less proliferative counterparts [52-55]. As gene regulation mechanisms mediated at the 3′UTRs are mostly repressive, it is generally assumed that a truncated 3′UTR will result in higher mRNA and/or protein levels [50]. It has been reported that 3′UTR shortening of oncogene mRNAs in cancer cells leads to increased protein abundance [54,56]. Intriguingly, the truncation of the ABCG2 3′UTR has also been reported in an undifferentiated human embryonic stem (HuES) cell line where its high ABCG2 expression was associated with the short 3′UTR variant forms [57]. In contrast, another differentiated HuES cell line with lower ABCG2 levels possesses a longer 3′UTR variant [57]. Sandberg et al. also found that rapidly proliferating cells express ABCG2 mRNA with shorter 3′UTRs, presumably to escape miRNA regulation [52].

#### ABCB1 (MDR-1/P-gp)

ABCB1 (MDR-1/P-gp) is the most extensively studied MDR transporter, which was discovered more than 30 years ago [58]. Overexpression of MDR-1/P-gp confers cancer cells resistance to a broad range of structurally and functionally diverse chemotherapeutic drugs [59]. It was proposed to express in more than 50% of all drug-resistant human tumors. Not long after the identification of the miRNAs regulating ABCG2, a few miRNAs directly repressing ABCB1 through binding to its 3′UTR (miR-27a, -451, -296, -298, -338, -1253) have been reported (Table 1; [14,26,27]). Downregulation of these miRNAs in resistant cancer cells leads to drug resistance.

#### ABCC1 (MRP-1)

Multidrug resistance-associated protein (ABCC1/MRP-1) transports a wide range of different drug classes and is also known to play a critical role in the development of MDR in cancer cells. Among the major MDR transporters, the regulation of MRP-1 by miRNAs is the least studied. To date, only miR-326 [29] and miR-1291 [30] were reported to modulate MRP-1 expression directly via interacting with its 3′UTR. In VP-16-selected MRP-1-overexpressing MCF-7 resistant cells, miR-326 was found to be downregulated and lead to MRP-1 overexpression. MiR-1291 has just been recently reported to mediate doxorubicin resistance in pancreatic cancer cells by targeting ABCC1 [30]. It was derived from a small nucleolar RNA (snoRNA: miR-1291/SNORA34), a new class of non-coding regulatory RNAs that is known to control the posttranscriptional modification of ribosomal RNAs [30]. The tissue or cell type specific processing of SNORA34 to miR-1291, and thus the overexpression of miR-1291, in pancreatic cancer may allow the development of tumor targeting therapy to combat MDR by selectively intervening the miR-1291 pathway.

#### ABCC2 (MRP-2)

MDR-associated protein 2 (MRP-2) is a unique ABC transporter that can mediate platinum (Pt) drug resistance [60]. Pt-based anticancer drugs, including cisplatin and oxaliplatin, are the mainstay of treatment for most solid tumors. ABCC2 can recognize GSH-conjugated form of Pt drugs and effectively pump them out of the cells to confer resistance. To date, only miR-297 has been reported to be down-regulated in a oxaliplatin-resistant colon cancer cell model (HCT116/L-OHP) to cause ABCC2 overexpression and Pt drug resistance [31]. A complementary binding site for miR-297 was identified on ABCC2 3′UTR to mediate the specific gene repression.

### Indirect regulation by miRNAs

#### MDR-1/P-gp

**Figure 2:**
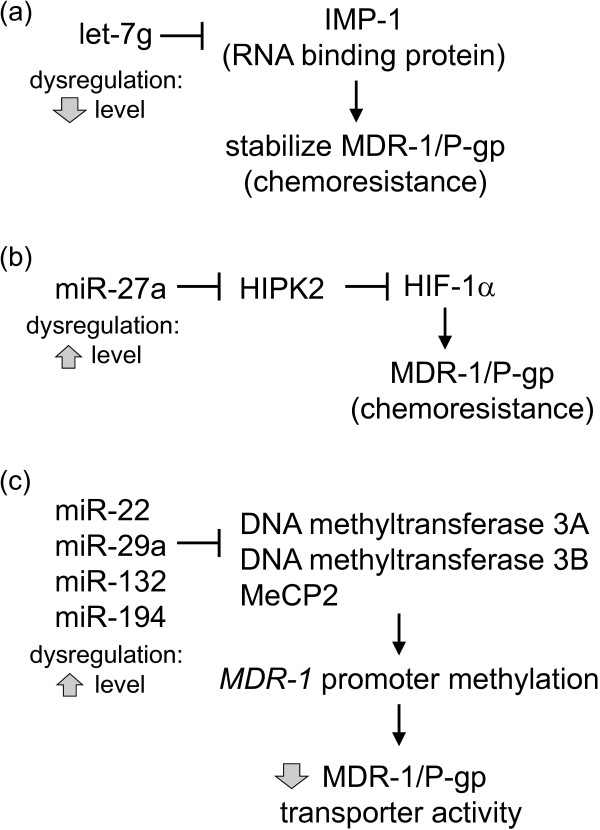
**Indirect regulatory mechanisms of MDR-1/P-gp by miRNAs. (a)** let-7 g downregulation is commonly observed in various cancers. It is known to target the RNA binding protein, IMP-1, which stabilizes MDR-1 mRNA. Therefore, let-7 g loss in resistant cells allows overexpression of IMP-1 and stability of MDR-1/P-gp to mediate drug resistance [28]. **(b)** Homeodomain-interacting protein kinase-2 (HIPK2) is a known target of miR-27a. HIPK2 has also been reported to inhibit HIF-1α. Increased expression of miR-27a in resistant cells leads to downregulation of HIPK2, which indirectly allows HIF-1α-mediated stimulation of MDR-1/P-gp and chemoresistance [23]. **(c)** A hypothetical miRNAs-DNA methylation machinery-*MDR-1* promoter methylation pathway. Increased expression of these miRNAs in resistant cells represses various DNA methylation mediators, thereby facilitating *MDR-1* promoter demethylation and increasing P-gp efflux activity to mediate chemoresistance.

Besides the various miRNAs discussed above that can directly modulate MDR-1/P-gp expression by interacting with complementary sequences at its 3′UTR, indirect regulation of the MDR transporter has also been reported. Let-7 g was reported to modulate acquired resistance of ovarian cancer to taxanes via IMP-1-mediated stabilization of MDR-1 [28]. IMP-1 is an RNA binding protein that stabilizes the mRNA of a number of target genes, including MDR-1 [61]. IMP-1 was known to be a validated target for let-7 g [62]. It follows that the loss of let-7 g commonly observed in various cancers [28] could allow the overexpression of IMP-1 and thereby stabilization of MDR-1/P-gp to mediate anticancer drug resistance (Figure 2a; [28]). Moreover, a novel miR-27a/HIPK2/MDR1/P-gp pathway has been proposed that lead to paclitaxel resistance in ovarian cancer cells [23]. Homeodomain-interacting protein kinase-2 (HIPK2) was reported to inhibit HIF-1α, thereby suppressing *MDR1* gene transcription and sensitize cancer cells to doxorubicin-induced apoptosis [63]. Therefore, increased expression of miR-27a in resistant cells leads to downregulation of HIPK2, which indirectly allows HIF-1α-mediated stimulation of MDR-1/P-gp and chemoresistance (Figure 2b; [23]).

Another noteworthy indirect mechanism for miRNA-mediated upregulation of MDR-1/P-gp involved the epigenetic alteration (i.e. hypomethylation) of the *MDR-1* promoter in resistant MCF-7/DOX cells (Figure 2c; [64]). The loss of cytosine methylation in the *MDR-1* promoter, which was shown to lead to P-gp overexpression and the resistance phenotype, was proposed to be mediated by the increased expression of miR-22, miR-29a, miR-132, and miR194. These miRNAs were known to target DNA methyltransferases 3A and 3B and methyl CpG binding protein 2, which mediate *MDR-1* promoter methylation [64-66]. Although the definitive proof for this hypothesis is still lacking, it has far-reaching implication in the etiology of MDR. A number of other key mediators of MDR (including ABCG2, BCL-2, PTEN, etc) are known to be repressed by DNA methylation, therefore aberrant increased expression of the aforementioned miRNAs in cancer cells may lead to derepression of these mediators to cause MDR.

#### ABCC3/ABCC6

**Figure 3:**
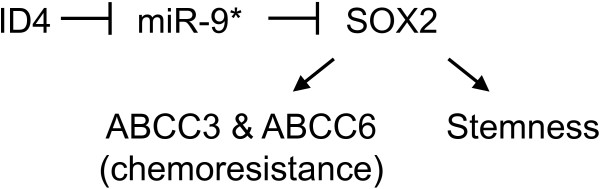
miR-9* is involved in an indirect regulatory mechanism of ABCC3 & ABCC6 to induce chemoresistance and stemness in glioma stem cells.

Unlike most ABC transporters that are highly expressed in various anatomic regions of the normal brain, ABCC3 and ABCC6 are not detectable in normal brain tissues [67]. Surprisingly, the specific role played by these two ABC transporters in the anticancer drug resistance of glioma stem cells has been recently reported [32]. A novel regulatory pathway Inhibitor of differentiation 4 (ID4)-miR-9*-SOX2-ABCC3/ABCC6 was proposed, which induces the stemness potential of glioma stem cells and chemoresistance (Figure 3). Of note, ABCC3 and ABCC6 are not direct targets for miR-9*. However, both of these ABC transporters are transcriptionally regulated by SOX2, which is elevated in glioma stem cells by ID4-mediated suppression of miR-9*.

## Regulation of MDR via non-transporter-mediated pathways by miRNAs

Alternations of several oncogenes (e.g. Bcl2, Ras, Src) and tumor suppressor genes (e.g. p53, RB, and p16) are closely associated with chemoresistance. However, the involvement of miRNA in these processes has just begun to be unraveled. A list of the most representative miRNAs regulating these non-transporter-mediated MDR pathways is summarized in Table 2. The list is by no means exhaustive but it aims to highlight a few examples according to the biological effect of the miRNA target gene (i.e. anti-apoptotic, drug metabolism, tumor suppressor, epithelial-to-mesenchymal transition (EMT)).

(a)  Anti-apoptotic

**Figure 4:**
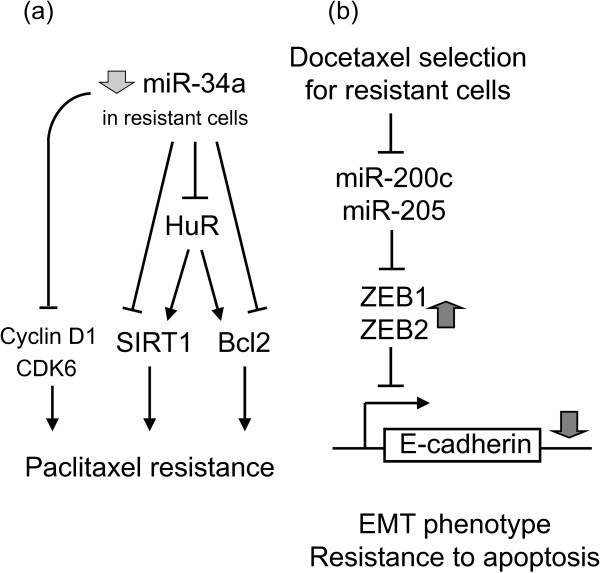
**miR-34a mediates paclitaxel resistance via both direct and indirect mechanisms. (a)** Schematic representation of direct and indirect mechanisms underlying miR-34a-mediated paclitaxel resistance. Direct pathway: miR-34a inhibit proliferation of paclitaxel-resistant PC3PR cells by directly suppressing the cell cycle regulators cyclin D1 and CDK6. Indirect pathway: miR-34a can enhance apoptosis by indirectly reducing expression of the anti-apoptotic gens (SIRT1 & BCL2) via modulating HuR [41]. **(b)** Indirect mechanism of miR-200c/-205-mediated EMT and resistance to apoptosis. Key signals that lead to EMT trigger expression of a variety of transcriptional repressors, including ZEB1, ZEB2/SIP1, Snail, and Twist. These repressors are the intracellular mediators of EMT by binding to E-box elements of genes, such as *CDH1*, that encode for the adhesion protein E-cadherin. After binding, they recruit histone deacetylases and other corepressors to facilitate transcriptional repression of E-cadherin. Reduced cellular E-cadherin expression subsequently will lead to loss of cell-cell adhesion and a series of other events, eventually leading to an invasive mesenchymal and drug resistance phenotype [43].

Most anticancer drugs work by induction of apoptosis. Alterations to susceptibility to apoptosis may lead to resistance to conventional cancer chemotherapy. *BCL2* (encoding the protein known as apoptosis regulator Bcl-2) is the most important pro-survival or anti-apoptotic factor often overexpressed in cancer and it is closely associated with chemotherapy resistance in various cancers. A number of miRNAs (including miR-15b, -16, -34a, -296, -1915) have been shown to modulate MDR by targeting BCL2 [19,26,40,41]. MiR-34a is of particular interest, where both direct and indirect regulatory pathways have been described (Figure 4a; [41]). MiR-34a can inhibit proliferation of paclitaxel-resistant PC3PR cells by directly suppressing expression of proteins involved in cell-cycle regulation such as CDK6 and cyclin D1. On the other hand, miR-34a has also been shown to enhance apoptosis by indirectly reducing expression of SIRT1 and Bcl2 via modulating HuR.

(b)  Drug metabolism

Dihydrofolate reductase (DHFR) is a key enzyme in cellular folate metabolism, which is the drug target of a widely used anticancer drug methotrexate. A naturally occurring SNP of DHFR (829C > T) has been identified near the miR-24 binding site within the 3′UTR of DHFR [20]. It interferes with miR-24 repression, thus resulting in DHFR overexpression and methotrexate resistance.

(c)  Tumor suppressor

A growing list of miRNAs has been identified to regulate PTEN, an important tumor suppressor, by binding to its 3′UTR [21,68,69]. For example, miR-214 was shown to interact with PTEN 3′UTR and inhibit PTEN translation, thereby leading to activation of the Akt pathway and cisplatin resistance [21]. The significance of this finding is that, once the miRNA-mediated regulation pathway is understood, apart from artificially altering the miRNA expression, the resistance phenotype can also be overridden by modulation of the upstream or downstream events (such as using either small-molecule Akt inhibitor or etopic expression of PTEN cDNA lacking 3′UTR) [21].

(d)  Epithelial-to-mesenchymal transition (EMT)

EMT is a cellular process that describes the change of an epithelial to a motile mesenchymal phenotype. Upon EMT, primary epithelial cancer cells acquire increased invasive and migratory abilities, eventually leading to metastases. Cancer cells selected for docetaxel resistance was found to exhibit EMT-triggered E-cadherin loss and reduced apoptosis [43]. Detailed mechanistic study revealed that these resistant cells have significantly reduced expression of miR-200c & -205. MiR-200c & -205 repress the transcriptional repressors, ZEB1/ZEB2, therefore reduced levels of these miRNAs allow more repression by ZEB1/ZEB2 and thereby downregulation of E-cadherin and EMT (Figure 4b; [43]). Another recent report by Liu et al. shown that miR-200c downregulation is frequently observed in metastatic melanoma, which is accompanied by overexpression of its target Bmi-1 (a Polycomb group protein class of transcriptional repressor) [42]. Upregulation of Bmi-1 was further shown to increase a number of MDR transporter genes (ABCG2, MDR1 and ABCG5) and to mediate loss of E-cadherin, collectively leading to a more resistant, malignant, and invasive EMT-like phenotype [42].

## Genetic polymorphism in miRNA binding sites and anti-cancer drug resistance

Regulation by miRNAs depends on the binding to seed sequences in the 3′UTR of their target mRNAs, which subsequently leads to degradation of mRNAs and/or translation blockade. Therefore, both sequence complementarity and thermodynamics of the binding determine the likelihood of interaction between miRNAs and their targets. It follows that sequence variations such as single-nucleotide polymorphisms (SNPs) in the miRNA target site (more specifically in the seed region) could affect the miRNA-mRNA interaction and subsequently expression of the miRNA targets. To this end, a recent bioinformatics study of the entire human genome revealed a relatively high frequency of miRNA target site SNPs that disrupt target sites or create new ones [70].

The most popular SNP occurring at miRNA target site, related to anticancer drug resistance, is probably the one identified by Mishra et al. on a miR-24 binding sequence that changed cell response to methotrexate [20]. The SNP (829C → T) near the miR-24 binding site in the 3′UTR of human dihydrofolate reductase (DHFR) was found to interfere with miR-24-mediated repression, thus resulting in DHFR overexpression and methotrexate resistance. Similar to this 3′UTR polymorphism, the phenomenon of alternative transcript polyadenylation (leading to shortening of 3′UTR) described above for the regulation of ABCG2 (Figure 1) [17] is another example where miRNA-mediated gene regulation can intersect with genetic variation to mediate anticancer drug resistance.

In fact, the study of 3′UTR variations is emerging as a new research avenue that holds promise for personalized pharmacotherapy [71,72]. The idea of “miRNA-pharmacogenomics” has been proposed by Bertino et al. that study the influence of genetic polymorphisms on miRNA function as a way to predict drug behavior and to improve drug efficacy [71]. Assessment of miRNA profile and 3′UTR polymorphisms in patients may thus allow personalized medicine [71].

## Use of miRNAs as prognostic markers for predicting chemotherapy response and/or chemoresistance: a rapidly growing field

Along with strong evidence suggesting that miRNAs can be employed clinically as biomarkers for cancer classification, diagnosis, and prognosis [7,73,74], miRNAs are also rapidly gaining popularity for predicting response to chemotherapy.

(a)  *In vitro* evidence from cell line studies

Blower et al. conducted one of the most systematic studies correlating global expression of 279 miRNAs and response to 3089 compounds in the NCI-60 drug screen cell line panel [15]. A good correlation between miRNA expression patterns and compound potency patterns was observed, thus strongly suggesting a prominent role played by miRNAs in mediating chemoresistance. When combined with gene expression and other biological data using multivariate analysis, miRNA expression profiles may help elucidate the complex mechanisms involved in chemosensitivity and chemoresistance.

(b)  Evidence from clinical specimen analyses (miRNAs expressed in tumors)

Recent data suggest that miRNA expression in patient samples at baseline could predict chemotherapy and radiotherapy response. A summary of these miRNAs is compiled in Table 3. A few representative ones are elaborated below.

**let-7i:** In epithelial ovarian cancer, let-7i expression was reported to be significantly reduced in chemotherapy-resistant patients with epithelial ovarian cancer (n = 69, p = 0.003) [84]. Follow up mechanistic investigation using both gain- and loss-of-function analysis revealed that reduced let-7i expression in ovarian and breast cancer cells are linked to resistance to cisplatin chemotherapy [84]. However, a contradictory finding about the role of let-7i in drug resistance has also been reported [13]. By modulating let-7i expression with pre-miRNA or miRNA inhibitor transfection in the NCI-60 panel of human cancer cell lines, let-7i inhibition was found to enhance the sensitivity of A549 cells to one of their investigational anticancer agent NSC670550 [13].

**miR-21:** miR-21 dysregulation has been reported to be a predictor of tumor response in pancreatic cancer patients to conventional cytotoxic chemotherapeutic agents, including gemcitabine, docetaxel, temozolomide and 5-fluorouracil [85,86].

**miR-26:** According to a miRNA microarray profiling study conducted in a relatively large cohort (n = 241) of patients with hepatocellular carcinoma, a list of tumor-related miRNAs closely associated with patient survival and treatment response to interferon was identified [81]. In particular, patients whose tumors had low miR-26 expression had shorter overall survival but a better response to interferon therapy than did patients whose tumors had high expression of the miRNA.

**miR-128b:** In a cohort of 58 non-small-cell lung cancer patients, miR-128b loss of heterozygosity (LOH) in the tumors was found to correlate with clinical response and prolonged survival following treatment with gefitinib [83]. Mechanistic investigation in NSCLC cell lines using miR-128b mimics or inhibitors confirmed that EGFR (the molecular target of gefitinib) is specifically regulated by the miRNA. LOH of chromosome 3p is a well-known and early genetic abnormality in lung cancer. Since miR-128b resides on the 3p22 locus, the aforementioned finding about the LOH of miR-128b could provide a functional link between a common genetic abnormality in lung cancer (i.e. loss of 3p22) and the frequent overexpression of EGFR in NSCLC patients. The clinical significance is that miR-128b may be used as a useful prognostic marker for selecting patient candidates to receive and benefit the most from EGFR tyrosine kinase inhibitors.

(c)  Circulating miRNAs as non-invasive biomarkers for predicting chemotherapy response

In recent years, circulating miRNAs, refering to the miRNAs present in cell-free body fluids such as plasma, serum, urine and saliva, etc, have been exploited for use as biomarkers in various disease states. These circulating miRNAs are bound to proteins including argonaute2 [88] and high density lipoprotein [89], or are encapsulated in exosomes [90], which render them highly resistant to RNase activity. Therefore, they can be reliably measured by the highly sensitive and relatively inexpensive method of quantitative polymerase chain reaction (qPCR). Moreover, the ease of access, minimal invasiveness and the possibiliy of repeated sampling of circulating miRNAs have also made them ideal candidates for use as biomarkers. Due to space limitation, we have highlighted a few representative circulating miRNAs that have been reported as useful predictive biomarkers for chemotherapy response (Table 4).

While a number of circulating miRNAs have been identified as useful biomarkers for predicting treatment response to chemotherapy and/or surgery, the detailed mechanism is usually not determined. It remains to be determined as to whether the circulating miRNAs are actively released by surviving cancer cells or derived from the dead cancer cells. Interestingly, in prostate cancer, Lucotti et al. has nicely demonstrated that cytotoxic treatment of DU-145 prostate cancer cells by fludarabine enhanced the release of a list of exosomes-associated prostate cancer secretary (PCS)-miRNAs, with the exception of miR-485-3p, which is retained by surviving cancer cells [97]. Follow-up mechanistic investigation revealed that the intracellular retention of miR-485-3p downregulate the transcriptional repressor NF-Y, and thus allowing the overexpression of drug resistance genes (including TOP2A, MDR1 and cyclin B2 pro-suvival genes) to mediate resistance [97].

In summary, tumoral miRNA expression at diagnosis may help predict the patients’ response to chemotherapy and also provide insights about mechanism of chemotherapy resistance. It may also provide guidance for rational and personalized chemotherapy selection. To this end, circulating miRNA as a novel prognostic or predictive tool is also rapidly gaining popularity [100,101] because of the non-invasive nature of the detection method. Moreover, miRNA-based profiling also has another added advantage over conventional mRNA-based methods. MiRNAs in formalin-fixed tissues, blood plasma and serum are known to be remarkably more stable than mRNAs to endogenous RNase digestion [102], thereby enabling their reliable extraction and analysis from patient specimens.

**Table 3:** Representative miRNAs from patient tumor specimens as predictive markers for treatment outcome in cancer therapy

**Cancer type**	**miRNA dysregulation**	**Chemotherapeutic outcome prediction**	**Reference**
Metastatic breast cancer	miR-26a ↑	- Multivariate analysis revealed that miR-26a and CDC2 (cell cycle regulator) are an optimal set of markers associated with favorable outcome on tamoxifen therapy, independently of traditional predictive factors (menopausal status, ER & PgR mRNA expression)	[75]
		- Mechanistic analysis showed that miR-26a repressed EZH2 to upregulate ER, thereby enhancing sensitivity to anti-estrogen therapy	
CRC	miR-181b↓ & let-7 g↓	Associated with responsiveness to 4^th^ generation fluoropyrimidine-based adjuvant therapy	[76]
CRC	miR-215↑	- miR-215 level generally downregulated in clinical CRC specimen	[77]
		- ↑miR-215 caused chemoresistance of HCT116 to methotrexate and tomudex	
		- No impact on treatment outcome from cisplatin and doxorubicin	
		- High level of miR-215 was found in CRC stem cells	
Metastatic CRC	miR-146b-3p↑ & miR-486-5p↑	- miRNAs level found to be more abundant in patients with mutant KRAS	[78]
		- Predictive of resistance to cetuximab (EGFR targeting monoclonal antibody)	
Metastatic CRC	miR-200b ↑	- In KRAS mutated tumors, ↑ miR-200b and ↓ miR-143 were associated with a good PFS in patients on cetuximab	[79]
	miR-143 ↓		
		- In wild-type KRAS patients, miRNA expression did not correlate with PFS in a multivariate model	
GBM	5-miRNAs signature (miR-181d, miR-518b, miR-524-5p, miR-566, miR-1227)	- Patients who had low risk scores from the 5-miRNA signature and received temozolomide treatment had better survival	[80]
		- Useful for identifying patients for more aggressive therapy	
Hepatocellular carcinoma	miR-26↓	Lower miR-26 is associated with shorter overall survival but a better response to interferon therapy	[81]
NSCLC	miR-21 ↑	Increased miR-21 in patients not responding to platinum-based chemotherapy	[82]
Lung cancer	miR-128b LOH	Predictive of clinical response and prolonged survival following gefitinib treatment	[83]
Ovarian cancer	let-7i↓	Predictive of resistance to cisplatin	[84]
Pancreatic cancer	miR-21↑	- Predictive of resistance to gemcitabine, docetaxel, temozolomide and 5-fluorouracil	[85,86]
		- Associated with shorter overall survival in the metastatic and adjuvant setting	
Pancreatic ductal adenocarcinoma	miR-10b ↓	Lower levels of miR-10b is associated with improved response to multimodality neoadjuvant therapy. Likelihood of surgical resection, delayed time to metastasis and increased survival	[87]

*Abbreviations:* CRC = colorectal cancer; EGFR = epidermal growth factor receptor; ER = estrogen receptor; GBM = glioblastoma multiforme; LOH = loss of heterozygosity; NSCLC = non-small cell lung cancer; PFS = progression-free survival; PgR = progesterone receptor.

**Table 4:** Representative circulating miRNAs reported to predict response to chemotherapy and/or surgery

**Cancer type**	**miRNA dysregulation associated with poor response**	**Sample type**	**Significance**	**Reference**
Breast	↑ miR-125b	Serum	Increased in patiens not responding to neoadjuvant chemotherapy	[91]
Breast	↑ miR-210	Plasma	Lower miR-210 plasma levels are associated with	[92]
			- complete response to trastuzumab (HER-2 targeted monoclonal antibody)	
			- surgical removal of tumor	
			- lack of tumor metastasis to lymph nodes	
Colorectal (CRC)	↑ miR17-3p	Plasma	- Elevated in both CRC tissue and plasma	[93]
			- Lower level detected in post-operative plasma is associated with responsiveness to surgery	
Colorectal (CRC)	↑ miR-29a	Serum	- Elevated in both CRC tissue and plasma	[93]
			- Help differentiate CRC from gastric cancer, inflammatory bowel disease and no tumor controls	
			- Lower level detected in post-operative plasma is associated with responsiveness to surgery	
Colorectal (CRC)	↑ miR-27b, miR-148a, miR-326	Plasma	Elevated in patients with metastatic CRC not responding to 5-fluouracil and oxaliplatin-based chemotherapy	[94]
Lung	↑ miR-21	Plasma	Increased; associated with resistance to platinum-based chemotherapy	[95]
Non-Hodgkin’s lymphoma (NHL)	↓ miR-92a	Plasma	- Remarkably lower in NHL patients (< 5%) than in healthy subjects	[96]
			- The very low plasma level of miR-92a increased in complete response phase but became lower again in the relapse phase	
Prostate	↑ Prostate cancer secretary (PCS)-miRNAs	Plasma/serum	- It is not clear whether circulating miRNAs are actively released by live cancer cells or derived from dead cancer cells.	[97]
			- *In vitro* cytotoxic treatment of DU-145 cells enhanced the release of exosomes-associated PCS-miRNAs, with the exception of miR-485-3p, which is retained by surviving cancer cells.	
			- The intracellular retention of miR-485-3p was shown to downregulate the transcriptional repressor NF-Y, thus allowing the overexpression of a few drug resistance genes (including TOP2A, MDR1, and cyclin B2 pro-survival genes)	
Prostate	↑ miR-21	Serum	- Increased in hormone-refractory prostate cancer	[98]
			- Associated with resistance to docetaxel-based chemotherapy	
Advanced renal cell carcinoma	↑ miR-192	Peripheral blood samples	- Models predicting poor and prolonged response to sunitinib were constructed	[99]
	↑ miR-193a-3p			
			- Ontology analyses revealed relevance to cancer-related pathways (angiogenesis and apoptosis)	
			- miRNA expression signatures may be used to identify patients who may benefit the most from 1^st^ line therapy with sunitinib	

## MiRNAs as druggable targets and miRNA-based therapeutics for circumvention of anticancer drug resistance

(a)  MiRNA-based therapeutics

Since miRNA expression is often dysregulated in cancer cells, approaches that modulate miRNA activity could potentially produce specific anti-cancer effect. With the advancement in technology, modulation of endogenous miRNA levels can now be achieved in several ways. Oncogenic miRNAs can be targeted for downregulation using various modified antisense oligonucleotides (also known as antagomirs) to their precursor or mature forms, whereas tumor suppressive miRNAs may be directly upregulated by using synthetic miRNA mimics (including siRNA-like oligoribonucleotide duplex and chemically modified oligoribonucleotide [103]) for an anti-cancer effect. In particular, antagomirs, with 2′-O-methylation or locked nucleic acid modifications, have drawn a lot of attention. Successful *in vivo* silencing of miRNA has been achieved by their systematic administration through tail vein injections into mice [104,105]. On the other hand, a few proof-of-concept studies using artificial synthetic miRNAs have been successfully performed to target a few oncogenes and produce anticancer effect [106-108]. A list of representative miRNAs as potential molecular targets for cancer therapy is compiled in Table 5.

(b)  Potential miRNA targets for resistance circumvention

Although the use of miRNAs for cancer chemotherapy has not yet been realized in clinical trials, it has recently been demonstrated in tissue culture systems that miRNA-targeted therapy may be useful in combination with conventional chemo-radiotherapy to sensitize the cancer cells. Table 6 summarizes a few representative miRNA-modulatory approaches to circumvent anticancer drug resistance. let-7 overexpression has been shown to confer radiosensitivity in lung cancer cell lines [123]. Inhibition of miR-21 and miR-200b was reported to enhance the sensitivity of cholangiocarcinomas to gemcitabine chemotherapy [120]. MiR-21 is of particular interest, which is overexpressed in most cancer types analyzed [124]. A landmark study has been reported to illustrate the phenomenon of “oncomiR addiction” in an *in vivo* model of miR-21-induced pre-B-cell lymphoma [119]. Most intriguingly, complete tumor regression can be achieved in a few days when miR-21 was inactivated by the antisense strategy *in vivo*. Given that aberrant miR-21 expression is known to reduce sensitivity of cancer cells to a number of anticancer drugs including tamoxifen, gemcitabine, doxorubicin and docetaxel [124], inactivating miR-21 may represent a novel strategy for cancer drug resistance circumvention. Most recently, a novel approach to deliver functional anti-miR-9 by mesenchymal stem cell-derived exosomes to glioblastoma multiforme (GBM) cells has been reported to circumvent P-gp-mediated resistance to temozolomide [112]. Site-directed targeted delivery of the anti-miR-9 to GBM cells was achieved because mesenchymal stem cells are able to migrate preferentially to the brain.

(c)  Therapeutic drugs altering miRNA profile in cancer cells

Apart from exhibiting aberrant expression of a few miRNAs, human cancers are in fact characterized by impaired miRNA processing and global miRNA dysregulation [125]. It has been recently shown that miRNA expression can be differentially altered by xenobiotic drugs in difference human cell lines [126]. The drugs identified are not necessarily anticancer drugs. The practical implication is that they could be safely administered with other conventional anticancer drugs in an attempt to reverse miRNA-mediated drug resistance. Along this line of investigation, the fluoroquinolone class of antibiotics has been shown to enhance RNA interference and promote miRNA processing [127,128]. This may represent a novel approach to modulate multiple miRNAs simultaneously or to restore the global miRNA expression (i.e. micRNAome) to provide a cancer-specific growth inhibitory effect. It has also been proposed that specific class of drugs might be screened for their effects on shifting the miRNA expression profile of a cancer cell towards that of a normal tissue [129]. To this end, the SM2miR database has been established to provide a fairly comprehensive respository about the influences of small molecules on miRNA expression, which could promote the futher development of miRNA-targeting therapeutics [130]. Calin et al. has proposed structure-based approaches (such as molecular docking) to identify compounds that may target specific miRNAs [131]. However, three-dimemsional structure prediction of miRNA still remains a challenge. There has also been attempts to identify small molecules targeting specific miRNAs in human cancers based on transcriptional responses [132]. As more is discovered in this research area, the specific modulation of miRNAs by therapeutic drugs may become feasible in the future.

(d)  Off-target effects

Each miRNA typically targets up to hundreds of transcripts directly or indirectly, and multiple miRNAs can target a given gene. It follows that miRNAs are also tied to some tightly regulated gene expression networks in normal cells. The therapeutic outcome of a miRNA-targeted chemotherapy or resistance reversal regimen may thus depend on the number of miRNA targets and the affinities for each of these targets that are expressed in a given tumor microenvironment. It could be difficult to rule out the by-stander off-target effects, if any. Moreover, an appropriate method to deliver the effective miRNA mimic/antagomir to the right cell type must also beconsidered in order to prevent unwanted side effects. Indeed, a fatal side effect as a result of saturation of miRNA pathways has been reported in an animal study [133]. It appears that these hurdles have to be overcome before an effective miRNA-targeted strategy can be realized for circumvention of anticancer drug resistance in cancer patients.

**Table 5:** MiRNAs as targets for cancer therapy

**Cancer type**	**miRNA target and its role in cancer**	Delivery system to modulate the miRNA in vivo*	**Reference**
Breast cancer	miR-34a – Tumor suppressor	Cationic liposomes	[109]
Glioblastoma	miR-145 – Tumor suppressor	Adenoviruses	[110]
Glioblastoma	miR-221-222 – Oncogene	Adenoviruses	[111]
Glioblastoma Multiforme (GBM)	miR-9 – promote expression of P-gp (a multidrug resistance efflux transporter)	- Mesenchymal stem cell-derived exosomes	[112]
		- To deliver anti-miR-9 to temozolomide-resistant GBM to reduce P-gp expression for resistance reversal	
Hepatocellular carcinoma	miR-26 – Tumor suppressor	Adenoviruses	[113]
Lung cancer	let-7 – Tumor suppressor	Adenoviruses	[114]
Lung cancer	miR-34a – Tumor suppressor	Cationic liposomes	[115]
Lymphoma	miR-155 – Oncogene	Polymer-based nanoparticles	[116]
Medulloblastoma	miR-17 ~ 92 cluster family – Sonic Hedgehog signaling	8-mer seed-targeting locked nucleic acid (LNA)-modified anti-miR oligonucleotides (nude mice)	[117]
Pancreatic cancer	miR-21 – Oncogene	Lentiviruses	[118]

* The miRNA target can be modulated by anti-miRNA oligonucleotides or miRNA-expressing constructs (delivered by viral or non-viral vectors).

**Table 6:** Novel approaches to circumvent chemoresistance by modulating unique miRNAs

**Cancer type**	**miRNA targeted for inhibition**	**Type of resistance circumvented**	**Delivery system for modulation of miRNAs**	**Reference**
B-cell lymphoma	miR-21 – oncomiR addiction	--	Antisense strategy	[119]
Cholangiocarcinomas	miR-21 & miR-200b	Gecitabine resistance mediated by PTEN-dependent activation of PI3K signaling	Transfection with miRNA-specific antisense oligonucleotides	[120]
Glioblastoma Multiforme (GBM)	miR-9 – indirectly promoting expression of the MDR transporter P-gp	P-gp-mediated resistance to temozolomide	- Mesenchymal stem cell-derived exosomes	[112]
			- To deliver anti-miR-9 to temozolomide-resistant GBM to reduce P-gp expression for resistance reversal	
GBM	miR-21	- Temozolomide	Transfection with anit-miR-21 oligonucleotide	[121]
		- Enhance apoptosis		
Lung cancer	miR-92b	Cisplatin resistance mediated by downregulation of the tumor suppressor gene *PTEN*	Transfection with anti-miR-92b oligonucleotide	[122]

## Conclusions

The emerging role of miRNAs as regulators of gene expression, and their dysregulation in human cancer has provided opportunities for their therapeutic application in the capacity of cancer detection, diagnosis and prognosis prediction. Selective targeting of some miRNAs may be useful in enhancing chemosensitivity, and may have future applications in modulating therapeutic response to molecular targeted chemotherapeutics in selected cancer subtypes. A better understanding about the complex regulatory pathways that control miRNAs function and their tumor-specific effect will be needed in order to fully realize the promise of miRNAs in cancer diagnosis, chemotherapy and drug resistance circumvention.

## Abbreviations

ABCG2/BCRP: Breast cancer resistance protein; ABC transporters: ATP-binding cassette transporters; CSC: Cancer stem cell; DHFR: Dihydrofolate reductase; EGFR: Epidermal growth factor receptor; EMT: Epithelial-to-mesenchymal transition; GSH: Glutathione; HuES: Human embryonic stem cell; LOH: Loss of heterozygosity; MDR-1/ABCB1: P-glycoprotein; MeCP2: Methyl CpG binding protein 2; miRNA/miR: microRNA; MRP-1/ABCC1: Multidrug resistance associated protein 1; PcG: Polycomb group protein; Pt: Platinum; PTEN: Phosphatase and tensin homolog; SnoRNA: Small nucleolar RNA; SP: Side population; 3′UTR: 3′untranslated region.

## Competing interests

The author declares that he has no competing interests.

## Author’s contributions

KKT completed the final draft and approved the manuscript.
